# Supplementary material for: Identification of photoperiod-induced specific miRNAs in the adrenal glands of Sunite sheep (Ovis aries)
Source: Front Vet Sci. 2022 Jul 22;9:888207. doi: 10.3389/fvets.2022.888207 (PMC9354845; doi:10.3389/fvets.2022.888207)
Supplement: Supplementary file 1 [file Data_Sheet_1.ZIP › Supplementary file/miRDeep2 file.zip!/miRDeep2 file/Novel_Structure/LP42A3/Novel_447.pdf]

Provisional ID	: NC_019466.2_11458
Score total	: 1.2
Score for star read(s)	: -1.3
Score for read counts	: -0.8
Score for mfe	: 1.8
Score for randfold	: 1.6
Score for cons. seed	:
Total read count	: 10
Mature read count	: 6
Loop read count	: 0
Star read count	: 4

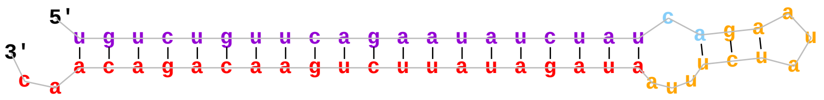

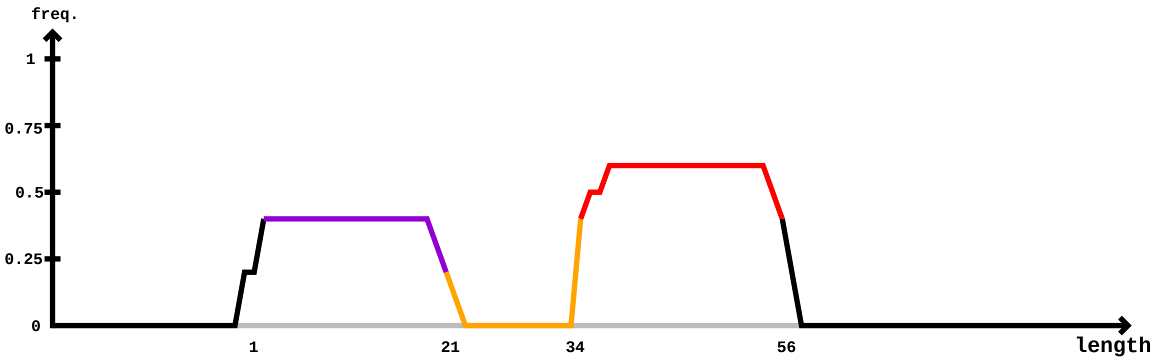


```
                           Star                          Mature
5'- acauuugcuccaucauagguugucuguucagaauaucuaucagaauaucuuuaauagauauucugaacagacaacaaaaaacccaaguaaaugaagcuacuaauucauuu  -3'  obs
    acauuugcuccaucauagguugucuguucagaauaucuaucagaauaucuuuaauagauauucugaacagacaacaaaaaacccaaguaaaugaagcuacuaauucauuu       exp
    .((((((((.........((((((((((((((((((((((.(((...)))...))))))))))))))))))))))..........))))))))................. reads   mm   sample
    ..................guugucuguucagaauaucu........................................................................ 1       0    seq
    ..................guugucuguucagaauaucua....................................................................... 1       0    seq
    ....................ugucuguucagaauaucuau...................................................................... 2       0    seq
    .....................................................auagauauucugaacagaca..................................... 1       0    seq
    .....................................................auagauauucugaacagacaa.................................... 1       0    seq
    .....................................................auagauauucugaacagacaac................................... 2       0    seq
    ......................................................uagauauucugaacagacaac................................... 1       0    seq
    ........................................................gauauucugaacagacaaca.................................. 1       0    seq
```
